# An assessment of the quality of life in hemodialysis patients using the WHOQOL-BREF questionnaire

**DOI:** 10.4103/0971-4065.45288

**Published:** 2008-10

**Authors:** B. S. Sathvik, G. Parthasarathi, M. G. Narahari, K. C. Gurudev

**Affiliations:** Department of Pharmacy Practice, J.S.S. College of Pharmacy, Mysore - 570 015, India; 1 Department of Nephrology, J S S Medical College Hospital, Mysore - 570 004, India

**Keywords:** Chronic renal failure, end stage renal disease, hemodialysis, quality of life

## Abstract

A cross-sectional study was conducted to evaluate the quality of life (QOL) of hemodialysis patients. An attempt was made to compare the QOL of hemodialysis patients with the QOL of the general population, renal transplant patients, and patients with a chronic disease, in this case, asthma. The WHOQOL-BREF questionnaire was used to assess the quality of life. Hemodialysis patients who had completed three months of maintenance hemodialysis (*n* = 75) were enrolled into the study. The quality of life of hemodialysis patients was found to be significantly impaired (*P* < 0.05) in comparison to healthy individuals of the general population, particularly with respect to the physical, psychological, and social relationship domains. In comparison to the quality of life of renal transplant patients, the quality of life of hemodialysis patients was significantly (*P* < 0.05) lower in all the four WHOQOL-BREF domains. Only in the environmental dimension was the quality of life of hemodialysis patients found to be significantly lower than that of the asthma patients. Female hemodialysis patients showed significantly (*P* < 0.05) lower quality of life than did male patients in the psychological and environmental dimensions of WHOQOL-BREF. A positive association was seen between higher education and the psychological functioning and the environmental dimensions of WHOQOL-BREF. Thus, the quality of life of hemodialysis patients was found to be considerably impaired when compared to that of healthy individuals of the general population as well as of renal transplant patients.

## Introduction

Over the past few decades, quality of life (QOL)^1^ research endpoints have emerged as valuable research tools in assessing the outcome of therapeutic intervention in chronic diseases.^2^ End stage renal disease (ESRD) is one such chronic disease causing a high level of disability in different domains of the patients’ lives, leading to impaired QOL.^3^,^4^

The availability of various renal replacement therapies (RRT) has reduced the severity of symptoms and resulted in longer survival of ESRD patients.^5^ Hemodialysis therapy is time-intensive, expensive, and requires fluid and dietary restrictions. Long-term dialysis therapy itself often results in a loss of freedom, dependence on caregivers, disruption of marital, family, and social life, and reduced or loss of financial income.^6^ Due to these reasons, the physical, psychological, socioeconomic, and environmental aspects of life are negatively affected, leading to compromised QOL.^7^

Due to cost constraints in India, patients often request for a reduction in the frequency of dialysis sessions, the use of less expensive dialyzers, dialyzer reuse, and do not typically receive erythropoietin therapy.^8^ Hence, augmenting the QOL may perhaps be a challenge and an observable fact of specific interest for renal health care teams. The concepts of QOL and quality-adjusted life years in chronic diseases are still emerging concepts in India. There are very few published studies dealing with this topic, especially in ESRD,^9^ and most of these are from the developed countries.^10^,^11^ The main objectives of our study were to assess the QOL in hemodialysis patients with reference to their physical, psychological, social, and environmental health dimensions, and to assess the effects of age, sex, income, level of education, duration of disease, co-morbidity, and treatment duration on the QOL of hemodialysis patients. An attempt was made to compare the QOL of hemodialysis patients with the QOL of healthy individuals from the general population, renal transplant patients, and patients with the chronic disease, asthma.

## Materials and Methods

Patients were recruited from dialysis centers of J.S.S Medical College Hospital and Basappa Memorial Hospital, Mysore. The criteria for inclusion were: ESRD patients who were aged 18 years and above of either sex; on regular twice a week hemodialysis for at least three months or more, or who had received renal transplant at least six months prior to enrollment into the study; able to speak/read the local language, Kannada, or English and be able to provide informed consent to participate in the study. Patients were excluded if they had malignancies, tumors or multiple organ system failure, major hearing impairment (inability to hear loud speech even with a hearing aid), rejection episodes, or any major surgical interventions in the previous three months. We chose patients who were on regular hemodialysis for at least three months before their enrollment into the study as QOL measurements are less likely to be influenced by metabolic instability and the mode of dialysis treatment after three months of maintenance hemodialysis.

To compare the QOL of ESRD patients with the QOL of patients suffering from another chronic ailment, asthma, patients of severity as per GINA (Global Initiative on Asthma) grades II, III, and IV were enrolled in the study. Healthy individuals were selected from the general population by conducting a health survey and on a voluntary basis. During the survey, participants were questioned about their illness. Only those patients without any history of illness were enrolled in the study. Ethical approval for the study was obtained from the Institutional Ethics Committee of JSS Medical College and Hospital.

### Instrument for Assessment of QOL

WHOQOL-BREF, a generic health-related questionnaire developed by the WHOQOL group was selected to quantify the health-related quality of life of ESRD patients. The WHOQOL-BREF consists of 24 facets and provides a profile of scores on four dimensions of quality of life: physical health, psychological, social relationships, and the environment. WHOQOL-BREF is available in both self-administered and interviewer-administered forms.

### Scoring the WHOQOL-BREF

The WHOQOL-BREF questionnaire was scored after its administration to the study subjects; the raw scores were converted to transformed scores. The first transformation converts scores to a range of 4–20 and the second transformation converts domain scores to a 0–100 scale. Higher scores reflect a better quality of life.

### Validation of the WHOQOL-BREF questionnaire

The WHOQOL-BREF questionnaire is available in 19 different languages including Kannada, the local language. The Kannada version of WHOQOL-BREF has been validated and has demonstrated good content validity, discriminate validity, test-retest reliability, and internal consistency.^12^

Hemodialysis subjects satisfying the study criteria were recruited from the two aforementioned study sites. Data were collected from each subject and documented in a suitably designed data collection form. The QOL of renal transplant patients was compared with the QOL of hemodialysis patients. The WHOQOL-BREF was administered to the healthy individuals from the general population (age- and sex-matched with the reference asthma and ESRD subjects) enrolled into the study to assess their QOL and compare it with that of hemodialysis patients. To check the hypothesis that all chronic disease conditions have an impact on the QOL of the patients, we compared the QOL of hemodialysis patients with that of patients with another chronic disease, asthma.

### Statistical Analysis

Statistical analyses were done using Windows version 11.0 of SPSS. Univariant relationships between sociodemographic (gender, working status, and residing area), ESRD-related variables (duration of dialysis, type of co-morbidity), and WHOQOL-BREF scores were analyzed with one-way ANOVA and Student's t-test. When performing ANOVA, if the omnibus F-test was found to be statistically significant, Tukey's pairwise, multiple comparison procedure was used for *post hoc* comparisons. Pearson's correlation was used to study the correlation between QOL scores of each domain of WHOQOL-BREF and continuous sociodemographic and kidney disease variables (age, literacy, income, education status, and co-morbidities). We conducted linear regression analysis to determine the strongest predictors of QOL. *P* < 0.05 was regarded as being statistically significant.

## Results

The demographic details of the hemodialysis patients (*n* = 75), renal transplant (*n* = 39), asthma patients (*n* = 35), and healthy individuals from the general population (*n* = 300) are presented in Table 1. Table 2 shows the clinical laboratory parameters of hemodialysis and renal transplant patients at the time of inclusion.

**Table 1:** Demographic characteristics of general healthy population sample, hemodialysis, transplant and asthma patients

Variables	Hemodialysis	Transplant recipients	Healthy subjects	Asthma patients
	(n=75)	(n=39)	(n=300)	(n=35)
Gender				
Male	58 (77.3)	35 (89.7)	198 (66.0)	20 (57.14)
Female	17 (22.6)	04 (10.2)	102 (34.0)	15 (42.86)
Age (years)				
< 30	17 (22.6)	16 (41.0)	108 (36.0)	06 (17.14)
31–60	45 (60.0)	20 (51.2)	160 (53.3)	27 (77.14)
> 60	13 (17.3)	03 (7.6)	31 (10.3)	02 (5.71)
Education level				
Illiterate	06 (8.0)	02 (5.1)	22 (7.3)	00 (00)
Up to 10th	39 (52.0)	06 (15.3)	115 (38.3)	04 (11.43)
Up to 12th	08 (10.6)	02 (5.1)	64 (21.3)	02 ((5.71)
Diploma	04 (5.3)	02 (5.1)	14 (4.6)	02 (5.71)
Degree	18 (24.0)	27 (69.2)	86 (28.6)	27 (77.14)
Marital status				
Married	53 (70.6)	22 (56.4)	166 (55.3)	19 (54.29)
Bachelor	18 (24.0)	17 (43.5)	98 (32.6)	16 (45.71)
Widower	04 (5.3)	Nil	36 (12.0)	Nil
Annual family income (Rs.)				
< 25,00	08 (10.6)	Nil	41 (13.6)	01 (2.86)
25,000–50,000	12 (16.0)	Nil	81 (27.0)	05 (14.29)
50,000–1,00,000	30 (40.0)	Nil	91 (30.3)	09 (25.71)
1,00,000–2,00,000	17 (22.6)	03 (7.6)	77 (25.6)	08 (10.67)
> 2,00,000	08 (10.6)	36 (92.3)	10 (3.3)	12 (34.29)
Employment status				
Working	28 (37.3)	21 (53.85)	233 (77.6)	14 (40)
Not working	40 (53.3)	17 (43.59)	22 (7.3)	18 (51.43)
Retired	07 (9.3)	01 (2.56)	45 (15.0)	03 (8.57)
Duration of receiving treatment (months)				
3–6	31 (41.3)	00	NA	06
7–9	10 (13.3)	11 (28.21)	NA	14
10–12	02 (2.6)	15 (38.46)	NA	10
13–24	24 (32.0)	10 (25.64)	NA	03
> 24	08 (10.6)	03 (7.69)	NA	02

NA: Not applicable, Numbers in parenthesis are percentages.

**Table 2:** Clinical laboratory values of hemodialysis and transplant patients

Laboratory parameter	Mean ± SD
Hemodialysis patients	
Hemoglobin (g/dL)	7.58 ± 1.34
Serum creatinine (g/dL)	7.49 ± 2.30
Serum urea (g/dL)	110.6 ± 33.45
Serum albumin (g/dL)	3.23 ± 0.31
Transplant patients	
Hemoglobin (g/dL)	11.96 ± 2.49
Serum creatinine (g/dL)	1.58 ± 0.68
Serum urea (g/dL)	48.75 ± 27.26
Serum albumin (g/dL)	3.9 ± 0.49

### Comparison of QOL scores

QOL of hemodialysis patients was found to be significantly (*P* < 0.05) impaired in comparison to the QOL of healthy individuals selected from the general population, particularly with respect to the physical, psychological, and social relationship domains, but not in the environmental domain. It was interesting to note that transplant patients reported significantly (*P* < 0.05) better QOL scores than did the healthy individuals in all domains, except physical health (*P* = 0.583) [Table 3].

**Table 3:** WHOQOL-BREF scores for the general healthy population sample, hemodialysis patients, transplant patients, and patients with asthma

Domains	Group	N	Mean ± SD QOL scores	*P* value
Physical health	HD	75	38.81 ± 17.1	< 0.001* (GHP vs HD)
	GHP	300	71.1 ± 14.2	0.583 (GHP vs TP)
	TP	39	78.22 ± 14.0	< 0.01* (TP vs HD)
	Asthma	35	46.13 ± 22.3	0.176 (HD vs Asthma)
Psychological health	HD	75	40.92 ± 18.6	< 0.001* (GHP vs HD)
	GHP	300	63.0 ± 13.6	0.03* (GHP vs TP)
	TP	39	73.9 ± 12.9	< 0.01* (TP vs HD)
	Asthma	35	45.7 ± 19.4	0.134 (HD vs Asthma)
Social relationship	HD	75	53.93 ± 16.9	< 0.001* (GHP vs HD)
	GHP	300	68.8 ± 14.6	0.001* (GHP vs TP)
	TP	39	81.5 ± 9.8	< 0.01* (TP vs HD)
	Asthma	35	55.13 ± 22.9	0.823 (HD vs Asthma)
Environmental	HD	75	60.5 ± 11.7	0.702 (GHP vs HD)
	GHP	300	61.26 ± 12.8	0.0009* (GHP vs TP)
	TP	39	79.4 ± 12.4	0.0007* (TP vs HD)
	Asthma	35	60.59 ± 11.73	0.009* (HD vs Asthma)

* *P* < 0.05 is considered as statistically significant, GHP: General healthy population, TP: Transplantation, HD: Hemodialysis

In hemodialysis patients, the highest QOL score was observed in the environment domain (60.59 ± 11.73) followed by social relationships (53.93 ± 16.91), psychological health (40.92 ± 18.66), and physical domain QOL scores (38.81 ± 18.36). Compared to renal transplant patients, hemodialysis patients scored significantly (*P* < 0.05) lower QOL scores in all four dimensions of the WHOQOL-BREF questionnaire [Table 3].

The comparison of the QOL scores of hemodialysis patients with those of asthma patients did not yield any significant differences (*P* > 0.05) in physical health, psychological health, and social relationships. Asthma patients reported a significantly (*P* < 0.05) higher QOL in the environmental domain than did the hemodialysis patients [Table 3].

### Association between demographic characters and QOL

Various demographic factors and their association to QOL were assessed in ESRD patients on maintenance hemodialysis; the findings are tabulated in Table 4. Data from 58 male and 17 female ESRD patients were selected for analysis. The female patients reported significantly (*P* < 0.05) lower QOL scores in the psychological (31.71 ±19.45) and environmental domains (54.57 ±15.61) compared to the male hemodialysis patients (43.50 ±17.7 and 62.28 ± 9.95 respectively) [Table 4].

**Table 4:** Sociodemographic and disease-related variables and QOL

Variable	N	QOL scores of WHOQOL-BREF domains			
		
		PF	PYF	SF	ED
Gender					
Male	58	39.64 ± 17.33	43.50 ± 17.7	43.50 ± 17.7	62.28 ± 9.95
Female	17	35.85 ± 22.11	31.71 ± 19.45	31.71 ± 19.45	54.57 ± 15.61
		df = 74	df = 74	df = 74	df = 74
		*P* = 0.500	*P* = 0.036*	*P* = 0.394	*P* = 0.029*
Working status					
Working	28	50.08 ± 16.15	51.66 ± 17.00	55.25 ± 15.72	65.58 ± 6.88
Not working	40	31.40 ± 16.54	32.77 ± 16.19	53.57 ± 17.82	56.34 ± 12.92
Retired	07	36.60 ± 15.32	46.40 ± 16.86	50.20 ± 18.83	66.40 ± 11.26
		F = 9.379	F = 9.579	F = 0.197	F = 5.862
		*P* = 0.000*	*P* = 0.000*	*P* = 0.0821	*P* = 0.005*
Residential area					
Local	39	43.50 ± 19.16	43.11 ± 20.06	51.50 ± 13.87	61.35 ± 11.29
Outsider	36	33.50 ± 16.11	38.43 ± 16.92	56.70 ± 19.68	59.73 ± 12.35
		df = 74	df = 74	df = 74	df = 74
		*P* = 0.029*	*P* = 0.320	*P* = 0.223	*P* = 0.586
Dialysis center					
JSS Hospital	58	40.00 ± 18.68	41.12 ± 17.97	63.75 ± 10.69	62.00 ± 5.65
BM Hospital	17	38.41 ± 18.43	40.85 ± 19.07	50.66 ± 17.41	60.12 ± 13.17
		df = 74	df = 74	df = 74	df = 74
		*P* = 0.768	*P* = 0.960	*P* = 0.006*	0.584
Duration of dialysis (In months)					
3–6	31	32.71 ± 16.63	34.28 ± 17.76	52.85 ± 19.05	54.82 ± 12.00
7–9	10	36.12 ± 14.93	36.12 ± 16.68	57.75 ± 08.08	61.00 ± 10.44
10–12	02	56.00 ± 18.77	56.00 ± 18.77	69.00 ± 16.26	69.00 ± 12.88
13–24	24	46.89 ± 18.52	46.89 ± 18.52	54.10 ± 15.42	66.62 ± 7.44
> 24	08	41.50 ± 21.84	41.50 ± 21.84	51.62 ± 20.94	60.59 ± 11.73
		F = 2.142	F = 2.701	F = 0.351	F = 3.972
		*P* = 0.087	*P* = 0.039*	*P* = 0.842	*P* = 0.006*
Primary kidney disease					
Hypertensive nephropathy	30	41.40 ± 16.93	42.60 ± 17.97	50.80 ± 14.67	59.90 ± 10.17
Diabetic nephropathy	10	31.33 ± 19.19	34.50 ± 20.80	67.66 ± 10.07	66.83 ± 10.81
HT and DM nephropathy	16	35.50 ± 17.37	41.25 ± 19.00	50.58 ± 24.57	61.08 ± 16.00
Glomerular nephritis	10	31.50 ± 14.05	36.87 ± 13.70	56.37 ± 16.10	58.87 ± 10.54
Others	07	47.00 ± 26.04	43.00 ± 25.72	58.00 ± 12.39	59.50 ± 13.11
		F = 1.228	F = 0.343	F = 1.576	F = 0.499
		*P* = 0.309	*P* = 0.848	*P* = 0.192	*P* = 0.736
Type of co-morbidity					
HT	38	39.97 ± 17.03	40.73 ± 18.24	52.63 ± 14.53	59.21 ± 10.94
DM	10	35.10 ± 19.03	44.50 ± 18.00	61.20 ± 11.70	68.20 ± 9.90
HT and DM	16	32.40 ± 17.95	32.60 ± 23.33	37.60 ± 33.26	54.00 ± 21.04
HT and HP	04	42.25 ± 19.70	40.50 ± 14.84	60.00 ± 20.19	64.00 ± 9.55
HP±HT±DM	01	19.00 ± 0.00	44.00 ± 0.00	44.00 ± 0.00	69.00 ± 0.00
HP±HT±IHD	02	28.50 ± 13.43	37.50 ± 17.67	69.00 ± 0.00	56.50 ± 9.19
Other	01	25.00 ± 0.00	19.00 ± 0.00	69.00 ± 0.00	50.00 ± 0.00
None	03	60.66 ±28.57	54.33 ± 31.89	52.33 ± 14.33	62.66 ± 6.50
		F = 1.150	F = 0.599	F = 1.542	F = 1.216
		*P* = 0.346	*P* = 0.754	*P* = 0.172	*P* = 0.310

PF: Physical functioning, PYF: Psychological functioning, SF: Social functioning, ED: Environmental domain, HT: Hypertension, DM: Diabetes mellitus, HP: Hepatitis, IHD: Ischemic heart disease

* *P* < 0.05 is considered as statistically significant, QOL: Quality of life

Employment status also influenced the QOL. There was a significant difference between QOL scores in physical health (*P* < 0.001), psychological health (*P* < 0.001), and environmental dimensions (*P* = 0.005) of hemodialysis patients with different employment status. *Post hoc* analysis showed that hemodialysis subjects who were employed, scored statistically significant higher scores in the physical (*P* < 0.001), psychological (*P* < 0.001), and environmental (*P* = 0.006) domains compared to hemodialysis subjects who were not employed [Table 4].

With respect to the influence of the duration of dialysis on the QOL, patients who were on dialysis for the last 10–12 months reported significantly better QOL scores in the psychological (*P* = 0.039) and environmental domains (*P* = 0.006) than did the patients with shorter and longer (than 10–12 months) durations of maintained hemodialysis [Table 4]. Patients from localities in Mysore reported significantly (*P* = 0.029) higher scores in the physical health domain compared to patients attending the clinic from towns/villages outside Mysore [Table 4].

There was no significant (*P* > 0.05) difference in the QOL scores of the two study centers with respect to the physical, psychological, and environmental domains. JSS hospital patients scored statistically significant (*P* = 0.006) higher QOL scores in the social relationship domain compared to BM hospital patients [Table 4].

Pearson's correlation showed a positive relationship between the annual family income and the physical, psychological, and environmental QOL scores. A similar observation was made between the education status of hemodialysis subjects and their psychological and environmental QOL scores. A positive relationship was also documented between the duration of dialysis and the psychological and environmental QOL scores. On the other hand, a negative association was observed between the number of co-morbidities and physical health. A statistically significant (*P* < 0.05) positive correlation was observed between the environmental dimension of WHOQOL-BREF and patients’ serum albumin and hemoglobin levels [Table 5].

**Table 5:** Pearson's correlation between continuous sociodemographic variables and disease-related variables with the WHOQOL-BREF dimensions of HD patients

Variable	Physical functioning	Psychological functioning	Social functioning	Environmental domain
Age	-0.179	-0.049	-0.046	0.144
Annual family income	0.263*	0.282*	-0.010	0.520*
Duration of dialysis	0.306*	0.351**	-0.002	0.443*
Education status	0.212	0.350**	-0.106	0.470**
Hemoglobin	0.182	0.137	-0.034	0.266*
Number of Co-morbidities	- 0.311*	-0.152	0.132	0.083
Serum albumin	0.164	0.175	-0.145	0.349**
Serum creatinine	0.054	-0.039	0.121	0.034
Urea	0.021	-0.111	0.043	-0.101

** : Correlation is significant at the 0.01 level (two-tailed)

* : Correlation is significant at the 0.05 level (two-tailed)

We used a linear regression model to determine the strongest predictors of QOL. After fitting the various demographic and disease-related variables into the linear regression model, we observed that the duration of dialysis, education, annual family income, the number of co-morbidities, and the hemoglobin and serum albumin levels were significant (*P* < 0.05) positive predictors of one or more dimensions of the WHOQOL-BREF [Table 6].

**Table 6:** Predictors of quality of life: Results from linear regression

Significant predictor	Beta	F	R^2^	*P* value
Physical health				
Duration of dialysis	+ 0.306	6.385	0.093	0.014
Income	+ 0.263	4.611	0.069	0.036
No. of co-morbidities	- 0.311	6.620	0.096	0.012
Employment status	- 0.314	6.711	0.098	0.012
Psychological health				
Duration of dialysis	+ 0.351	8.723	0.123	0.004
Education status	+ 0.350	0.122	8.651	0.005
Gender	- 0.263	4.611	0.069	0.036
Income	+ 0.282	5.376	0.080	0.024
Social functioning				
No significant predictors for this dimension of QOL				
Environmental health				
Albumin	+ 0.380	10.460	0.144	0.002
Duration of dialysis	+ 0.443	15.123	0.196	< 0.001
Education status	+ 0.470	17.592	0.221	< 0.001
Gender	- 0.274	5.021	0.075	0.029
Hemoglobin	+ 0.271	4.898	0.073	0.031
Income	+ 0.520	23.000	0.271	< 0.001

^*^ *P* < 0.05 is considered to be statistically significant

Gender and employment status were significant negative predictors of psychological (*P* = 0.036) and environmental dimensions (*P* = 0.029). Employment status was observed to be a significant negative predictor (*P* = 0.012) of the physical health dimension of WHOQOL-BREF [Table 6]. However, variables such as age, marital status, the type of co-morbidity, and the primary cause of the kidney disease were not associated with any of the QOL dimensions of WHOQOL-BREF [Tables 4 and 5].

## Discussion

QOL is becoming an important outcome measure after the initiation of renal replacement therapies. The major therapeutic goal is to improve the functioning ability of these patients so that they can enjoy life to its fullest possible extent. This study's results illustrate how physical, psychological, social functioning, environmental, and general health were affected in ESRD patients.

Although QOL scores in hemodialysis patients were significantly low in the physical, psychological, and social domains compared to those in the healthy subjects, there was no significant (*P* > 0.05) difference between the QOL scores of both these groups in the environmental domain. The low physical health scores in hemodialysis patients clearly demonstrate that daily activities were disturbed in ESRD patients as they were more dependent on the renal replacement treatment for their survival. Similar observations have been reported from studies comparing the QOL scores in chronic renal failure patients undergoing hemodialysis to general healthy population samples.^13^,^14^ In contrast to this, a few investigators have reported similar QOL for chronic renal failure patients and healthy individuals.^15^,^16^

It is interesting to note that although the majority of our study patients did not have adequate financial security and suffered a loss of income while being on hemodialysis, their environmental domain scores were not significantly lower than those of the healthy individuals in the study. Most of the study patients revealed that they had enough time for their recreation/leisure activities and a good home/physical environment. The patients were satisfied with their access to health services, yet another contribution to the absence of any significant (*P* > 0.05) difference in the QOL scores. However, the study was carried out in dialysis centers where the authors worked and therefore, the patients’ loyalty to the medical team may have made them answer the questions in positive ways, resulting in no significant differences in their QOL scores.

The overall QOL of employed hemodialysis patients, was substantially better than that of the retired and the unemployed groups. Employed patients scored better in their physical, psychological, and environmental health domains. The findings of our study are consistent with those of other studies that reported better QOL scores in employed patients in the physical functioning, mental health, and social functioning domains.^17^–^19^ Financial independence, to some extent, might have contributed to the higher QOL scores in the employed group. In addition, better mobility, work capacity, and less restriction in daily activities are possible factors contributing to the better QOL scores in the aforementioned domains. Employment has been found to be a vital factor improving the QOL of ESRD patients.^20^ However, a study conducted by Juergensen *et al*. did not find any difference in the QOL of employed and unemployed hemodialysis subjects.^21^

The environment does play a major role in determining health status. The environmental domain assesses the influence on the QOL of factors such as financial resources, the work environment, access to health and social care, freedom, security, and participation and opportunities for leisure activities. Despite the fact that ESRD patients did not have enough money or financial security for their treatment, the scores in the environmental domain were not significantly (*P* < 0.05) lower than the corresponding ones of the healthy subjects. During QOL assessment, most of the patients expressed that they had enough time for their recreation/leisure activities and good home/physical environments. The hemodialysis patients were satisfied with their access to health care services, further contributing to the lack of any significant (*P* > 0.05) difference in the QOL scores compared to those of the healthy subjects.

The level of school education was associated with two dimensions of WHOQOL-BREF. Subjects with higher education reported significantly higher QOL scores in the environmental dimension. The results of our study are consistent with findings of previous studies that reported a positive relationship between the level of school education and the QOL.^17^,^22^ A higher school education is known to play an essential role in raising the awareness of chronic diseases and in a better coping ability with chronic disease.^23^

The role of higher income is reflected in the higher scores in all domains of QOL, except for the social relationship domain. Our study results are consistent with findings of other studies that reported a positive association between family income and QOL scores.^17^,^22^ The higher income of an individual improves the ability of the patient to afford the required treatment and ensures a better QOL. A secure income is a reassurance to the patients and contributes to their psychological wellbeing. Financial difficulties due to premature retirement or loss of employment due to the disease may result in deterioration of QOL.

Patients from localities of Mysore reported significantly (*P* < 0.05) higher scores in the physical domain compared to semiurban and rural patients. As patients from outside Mysore had to travel a distance for dialysis, they spent more energy, money, and time in traveling.^24^ This may have led to a restriction in the daily activities, at least on the days they came for dialysis, contributing to the lower scores in the physical domain.

We did not observe any influence of the type of co-morbidities or the type of primary kidney disease on the QOL of hemodialysis subjects. A few studies have reported diabetes as a co-morbidity of ESRD resulting in significantly lower QOL scores.^25^,^26^ However, a negative relationship was observed between physical functioning and the number of co-morbidities. Our finding is consistent with other studies that observed a negative relationship between co-morbities and the QOL.^17^,^27^ An increase in the number of co-morbidities may worsen the QOL of patients due to physical, psychological, and emotional reasons.^17^

We observed lower scores in psychological and environmental domains in female subjects compared to the males. The majority of female patients felt that they were a burden to their families and were apprehensive about their bodily image and appearance. This might have contributed to the lower QOL scores in the environmental and psychological domains in female ESRD subjects. Other investigators have also reported lower health-related QOL in women than in men.^28^,^29^ However, the exact cause for the lower QOL in female ESRD patients is not clear. But it is possible that factors such as biological or cultural factors and biases in the provision of care or differences in the physicians’ attitude towards female patients might have contributed to the lower QOL scores.^30^,^31^

Many of the patients were dissatisfied with themselves and they often had negative feelings such as anxiety, melancholia, depression, and hopelessness. Most of them felt that they were a burden to their families. This resulted in lower scores in the psychological domain of ESRD patients than in the healthy subjects. The majority of study subjects, especially males, were not satisfied with their sex lives and they admitted decreased interest or no interest in sex. Similar findings have been reported in both males and females after the initiation of dialysis.^32^,^33^

We observed a positive relationship between hemoglobin and albumin levels of hemodialysis patients with the environmental QOL dimension; several studies have documented similar observations.^34^,^35^ Low hemoglobin and albumin levels are known to cause a negative impact on the QOL of hemodialysis patients.^34^,^35^ The hemoglobin and serum albumin levels of our study patients remained below the target level of 11 g/dL and 4 g/dL respectively.^34^,^36^ This observation suggests an urgent need for interventional strategies to elevate hemoglobin and albumin levels to their target levels.

One of the objectives of our study was to compare the QOL of hemodialysis patients with that of renal transplant patients, healthy individuals, and patients with another chronic disease, asthma. To achieve this objective, we used the WHOQOL-BREF, a generic questionnaire, whose wide applicability, language validity, and environmental domain make it relevant for this study. Moreover, such generic instruments can be administered to both diseased and healthy subjects.

Conflicting reports have been published in this area of comparison of the QOL of hemodialysis patients with that of the general (healthy) population. A number of reasons could account for the differences in these contradictory findings such as the age of the patients, the sample size, lack of prospective and longitudinal studies, the QOL instruments used, which may have been analogous but not similar. In addition, the QOL of the healthy general population itself may be low in developing countries compared to that of the healthy population in developed countries.

Compared to transplant patients, hemodialysis patients scored significantly (*P* < 0.05) lower QOL scores in all the four domains of WHOQOL-BREF questionnaire. These findings are similar to those of earlier published reports where higher scores were observed in the physical, social, and emotional domains of transplant patients than in hemodialysis patients.^37^,^38^ Consistent with earlier reports, we observed better psychological, social relationship, and environmental QOL in renal transplant patients than in the healthy subjects.^39^,^40^ This is probably due to a belief of the patients in considering a successful kidney transplant as a panacea. A successful kidney transplant has a positive impact on perceived health status and brings forth promises of an extended, enhanced QOL and a sense of personal liberation by raising the self-esteem due to the empowerment bestowed. A live-related kidney transplant would reinforce and intensify the emotional bondage between the recipient and a supportive family, leading to better psychological well being of the recipient.^41^,^42^

For chronic disease comparisons, we compared the QOL of hemodialysis patients with that of asthma patients because of the easy availability of the asthma patients in our study center. Previous comparative studies did not show any significant difference in the QOL of hemodialysis patients compared to that of patients with other chronic diseases such as congestive heart failure, rheumatoid arthritis, or chronic lung disease.^43^,^44^ Chronic diseases are reported to have an impact on the QOL. However, the extent of impact may be different for different chronic diseases as observed in this study.

The main limitation of our study was that all our study patients were undergoing twice-a-week dialysis instead of thrice-a-week dialysis due to economic constraints. This reduced frequency is known to limit the QOL of dialysis patients and made it difficult to compare this study's findings with those of other studies, where patients were undergoing thrice-a-week dialysis session or daily dialysis of short durations. This study was carried out in dialysis center where the authors worked, which could have influenced the patients to positively answer the questions pertinent to disease-related aspects in the WHOQOL-BREF questionnaire. In addition, most measures were self-administered questionnaires that may be influenced by fluctuations in the respondent's attention, motivation, comprehension, and response biases such as social desirability, which can potentially cause measurement error.^30^

The results of this study suggest that the QOL of hemodialysis patients is considerably impaired compared to that of the healthy subjects, especially with respect to the physical, psychological and social relationship domains. Renal transplant patients have better QOL in all the four dimensions of the WHOQOL-BREF compared to hemodialysis patients.
